# Biomimetic Mineralized Hydrophilic Polyurethane Primers for Inducing Dentin Tubule Fillings

**DOI:** 10.3390/polym14214716

**Published:** 2022-11-03

**Authors:** Zilu Tian, Shiyang Yu, Huimin Wang, Yubin Yang, Xuanyan Zhu, Song Zhu

**Affiliations:** Department of Prosthetic Dentistry, Hospital of Stomatology, Jilin University, Changchun 130012, China

**Keywords:** mineralization, etch-and-rinse, dental restoration, peptide

## Abstract

This experiment aimed to synthesize a biomimetic mineralized hydrophilic polyurethane dentin primer containing DDDEEKC peptide (DDDEEKC-PU) to fill dentin tubules and induce mineralization. The degree of conversion (DC) was tested. Dentin samples were acid-etched and treated with DDDEEKC-PU. They were immersed in stimulated human fluid (SBF) for 7, 14 and 28 days. Dentin permeability, X-ray diffraction (XRD), field emission scanning electron microscopy (FESEM) and Vickers hardness were measured. After 28 days, regenerated minerals were deposited on resin tags which were confirmed to be hydroxyapatite (HAp). The minerals reduced the dentin permeability and improved the microhardness. DDDEEKC-PU was able to fill dental tubules immediately and induce mineralization simultaneously.

## 1. Introduction

Dentin is the primary component of teeth, consisting of 70% hydroxyapatite (HAp), 20% type I collagen fibrils and 10% plasma-like dentin tubule fluid. Dentin tubules are oriented from the pulp cavity crossing the whole layer. Inside each tubule lies nerve branches and circulates dentin tubular fluid, which flows outward from the pulp cavity under positive pulp pressure [1]. When dentin tubules are exposed, the tooth experiences a short period of severe pain and suffers from dentin sensitivity upon external stimulation [2].

Exposed tubules cause the extravasation of dentin tubular fluid, contaminating the bonding interface during dentin bonding and blocking the hydrophobic adhesive resin from permeating the determined region and making it difficult to form a structurally homogeneous and stable hybrid layer [3,4]. The unbound water acts with inherent caries or acid-etched activated hydrolases, such as metalloproteinases and cysteine proteinases, accelerating the enzymatic hydrolysis of nude type I collagen. They destroy specific peptide bonds, disintegrate the peptide chains into soluble fragments and ultimately break the hybrid layer [5]. Unbound water also interferes with the polymerization of the adhesive and accelerates the plasticization of polymer molecules. The unpolymerized residual monomers speed up polymer molecule softening, mediate the oxidative stress response and cause pulpal symptoms [6,7]. Therefore, the unbound water significantly harms dentin bonding.

Biomineralization is a natural and progressive method of dehydration [8]. This experiment seeks to seal dentin tubules and induce nascent apatite crystal deposition by dentin primers to replace the unbound water. In the etch-and-rinse adhesive system, the primer is mainly composed of hydrophilic functional monomers and hydrophobic cross-linking monomers to penetrate the demineralized collagen network. Then, adhesive resin monomers connect with primers to replace water and encapsulates the type I collagen to obtain micromechanical interlacing. The primer, which acts as a bridge between the dentin and adhesive resin, must have improved characteristics to enhance the strength of the adhesive system [9,10]. Non-collagen proteins (NCPs) are rich in acidic amino acids, such as glutamic acid and aspartic acid, which carry a large number of negative charges. Therefore, researchers have proposed that NCP analogs can be used to direct the natural mineralization process [11]. Statherin, a 45-amino acidic protein in saliva, is secreted by parotid and submandibular glands. The 15 amino acid N-terminal of statherin (DpSpSEEKFLRIGRFG, SN15, D: Aspartic acid; pS: Phosphorylated serine; E: Glutamic acid; K: Lysine; F: Phenylalanine; L: Leucine; R: Arginine; I:Isoleucine; G: Glycine) is the bioactive site for remineralization via Ca^2+^ chelation. The phosphorylated serines are replaced with aspartic (DDDEEKFLRIGRFG, SN_A_15) to avoid post-translational modification but maintain Ca^2+^ affinity. Researchers reported that the first six-peptide fragments (DDDEEK) had high negative charge density and helical conformation. These characteristics endow DDDEEK with increased affinity for Ca^2+^ and PO_4_^3−^ [12,13,14].

Since the 1990s, polyurethane (PU) has been widely used in drug sustained-release carriers, cartilage and bone repair materials due to its excellent biocompatibility and modifiability [15,16,17]. Studies have shown that it can form stable chemical bonds with HAp and complex resins given its highly unsaturated free groups, reactive isocyanate (-NCO) and carbamate (-NHCOO-). Previous researchers found that the PU adhesive was suitable for direct dental restoration. The polyol hybrid acrylate-modified PU dentin adhesive exhibits flexible bonding with dentin and buffers various external stresses. It had good hydrolysis resistance and thermal stability, effectively reducing microleakage at the bonding surface. Gong et al. (2019). synthesized photopolymerizable and moisture-curable PU, which formed a stable and durable chemical bond with dentin and resin restoratives [18].

The aim for this paper was to synthesize light-curable hydrophilic PU and formulate a biomimetic mineralized hydrophilic polyurethane dentin primer. The null hypotheses tested were (1) dentin tubules cannot be filled with the novel dentin primer and (2) the novel primer cannot induce mineralization.

## 2. Materials and Methods

### 2.1. Materials

Poly(tetrahydrofuran ether glycol) 1000 (PTMEG 1000), 2-hydroxyethyl methacrylate (HEMA), triethylene glycol dimethacrylate (TEGDMA), dibutyltin dilaurate (DBTDL), acrylic acid (AA), isophorone diisocyanate (IPDI), 2,2-dihydroxymethylpropionic (DMPA), γ-methacryloxypropyl trimethoxy silane (*γ*-MTS), 2,2-dihydroxymethylpropionic, triethylenediamine, camphorquinone (CQ), ethyl-4-dimethylamino-benzoate (EDMAB), acetic acid and *n*HAp were purchased from Sigma Aldrich Corp, St. Louis (MO, USA). Tetrahydrofuran (THF) was supplied by Tianjin Tiantai Refine Chemicals Co., Ltd. (Tianjin, China). Peptide DDDEEKC was obtained from ChinaPeptide (Shanghai, China). Simulated body fluid (SBF) was obtained from Beijing Leagene Biotechnology Co., Ltd. (Beijing, China). Potassium hydroxide was supplied by Beijing Chemical Co. (Beijing, China).

### 2.2. Synthesis of Light-Curable Hydrophilic PU

PTMEG 1000, THF and HEMA were thoroughly dewatered before use. The reaction was performed in a three-necked round-bottom flask with mechanical stirring, condensation reflux and a water-cooled condenser at 68 °C. Fourier infrared spectroscopy (FTIR) (Nicolet iS 10, Madison, USA) analysis of the products at each phase was performed using the coated film method with potassium bromide tablets as the background.

**Figure 1:**
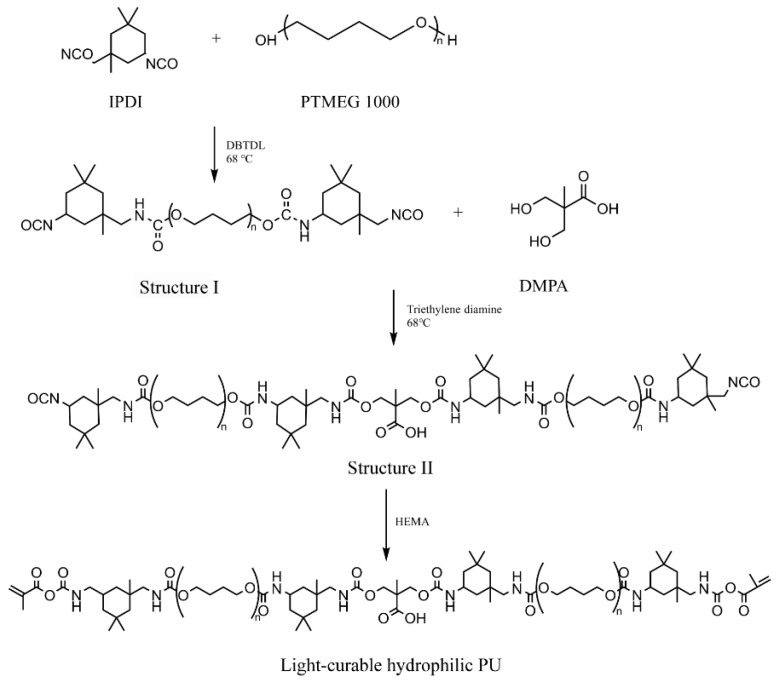
The main reaction synthesis of the light-curable hydrophilic PU. DBTDL, dibutyltin dilaurate (the catalyst); IPDI, isophorone diisocyanate; HEMA, 2-hydroxyethyl methacrylate; PTMEG 1000, poly(tetrahydrofuran ether glycol) 1000; DMPA, 2,2-dihydroxymethylpropionic.

IPDI (4 g, 0.018 mol) and DBTDL (0.0001 g, 0.3%) were mixed in 20 mL THF and transferred into a three-necked flask. PTMEG 1000 (6 g, 0.006 mol) was dissolved in 50 mL THF and then added dropwise. When the -OH at 3400 cm^−1^ could not be observed via FTIR, 1.6 g (0.012 mol) DMPA and triethylenediamine were added. When the signal intensity of -NCO at 2270 cm^−1^ decreased, 1.56 g of HEMA (0.012 mol) was added, and the reaction continued for 12 h. The solvent was removed under vacuum with a rotary evaporator at 40 °C. The main reaction process described above is shown in Figure 1.

### 2.3. Synthesis of Water Absorption Agent

A saturated aqueous solution of potassium hydroxide was configured. It was slowly added to AA to obtain AA with a neutralization degree of 60% (AA_60%_). The reaction was performed in an ice-water bath with magnetic stirring.

### 2.4. Nanohydroxyapatite Modified by γ-MTS

**Figure 2:**
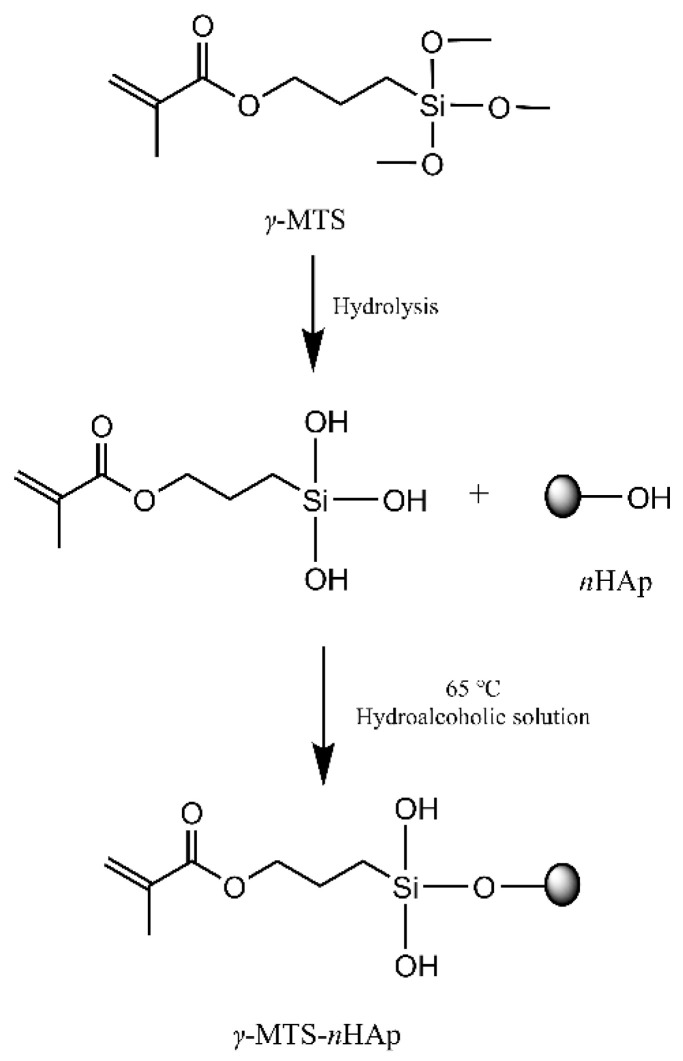
The primary reaction process of nanohydroxyapatite modified by *γ*-MTS.

*γ*-MTS was diluted with water and methanol (water/methanol/*γ*-MTS = 1:9:5). After hydrolysis for 30 min at room temperature, 5 g *n*HAp was dispersed in 50 mL *γ*-MTS hydrolysis solution using ultrasonic agitation. Then, the suspension was transferred into a three-necked flask and mechanically stirred at 65 °C for 2 h. A vacuum with a rotary evaporator removed the solvent at 70 °C. The products were washed five times and dried at 80 °C. The primary reaction process described above was shown in Figure 2.

### 2.5. Formulation of Biomimetic Mineralized Hydrophilic Polyurethane Dentin Primer

Briefly, 2.1 g light-curable hydrophilic PU, 0.45 g HEMA, 0.45 g TEGDMA, 0.53 g AA_60%_ CQ/EDMAB 0.01/0.024 g and 0.36 g *γ*-MTS-*n*HAp were mixed well using the Speedmixer TM (FlackTek, South Carolina, USA). Then, biomimetic mineralized hydrophilic polyurethane dentin primers with peptide DDDEEKC contents of 0 mg/g (0-PU), 3 mg/g (3-PU), 5 mg/g (5-PU) and 7 mg/g (7-PU) were configured.

### 2.6. Determination of DC

The DC of each group was estimated using FTIR (VERTEX-80V, Germany) spectroscopy at a wavelength of 400–4000 cm^−1^, resolution of 4 cm^−1^ and 32 scans. The samples were coated on potassium bromide press sheets and the uncured spectra were first collected. Each sample was light-cured for 20 s (light intensity 1000 mW/cm^2^) (SLC-VIIIA) and the spectra were collected again (n = 6). The DC was calculated using the following formula:

$$DC\%=\left[1-\frac{{\left(\frac{A_{1640}}{A_{1720}}\right)}_{cured}}{{\left(\frac{A_{1640}}{A_{1720}}\right)}_{uncured}}\right]\times 100\%$$

### 2.7. Preparation of Dentin Specimens

Non-carious human third molars were obtained from patients of certain ages (18 < age < 30 years) after approval by the Ethics Committee for Human Studies of the School and Hospital of Stomatology, Jilin University, China (2021 NO. 83 on 21 January 2021). The specimens were stored in 0.1% thymol solution at 4 °C for no longer than 3 months. The enamel and roots (2 mm below the enamel–dentin junction) were removed using a low-speed diamond saw (SYJ150, Kejing, China) under running water cooling to obtain dentin discs (10.0 × 8.0 × 1.0 mm). The dentin discs were polished on 600-, 800-, 1000-, 1500- and 2000-grit silicon carbide papers under running water and ultrasonically cleaned twice for 10 min. Then, the discs were etched with 37% phosphoric acid gel for 15 s, rinsed thoroughly for 30 s and lightly blown for 20 s to obtain the demineralized layer.

Then, dentin discs were randomly divided into different experimental groups. They were treated with DDDEEKC-PU for 20 s, slightly air-dried for 10 s and light-cured for 20 s. The prepared samples were embedded in 5 mL SBF, which was changed every 7 days, and mineralized at 37 °C for 7, 14 and 28 days.

### 2.8. Characterization of Remineralization

#### 2.8.1. Vickers Microhardness Measurements

The dentin surface microhardness was tested using a Vickers hardness tester (HMV-G20, Shimadzu, Japan). Three points were randomly assessed in each sample (n = 5). The maximum load was 1 N, which was held for 30 s.

#### 2.8.2. XRD

The surface crystalline components were analyzed by XRD (Rigaku Dmax, Japan). The operating parameters of XRD were as follows: Cu K-α target radiation (λ = 0.1542 nm); tube current, 40 mA; tube voltage, 40 kV; scanning speed, 2° min^−1^ (each step: 0.05°, 40 s/step) and scanning range, 25–50°. The XRD spectra were analyzed using Jade 6.5 and the full width of the half-peak (FWMH) was analyzed for each group.

#### 2.8.3. Dentin Permeability Evaluation

**Figure 3:**
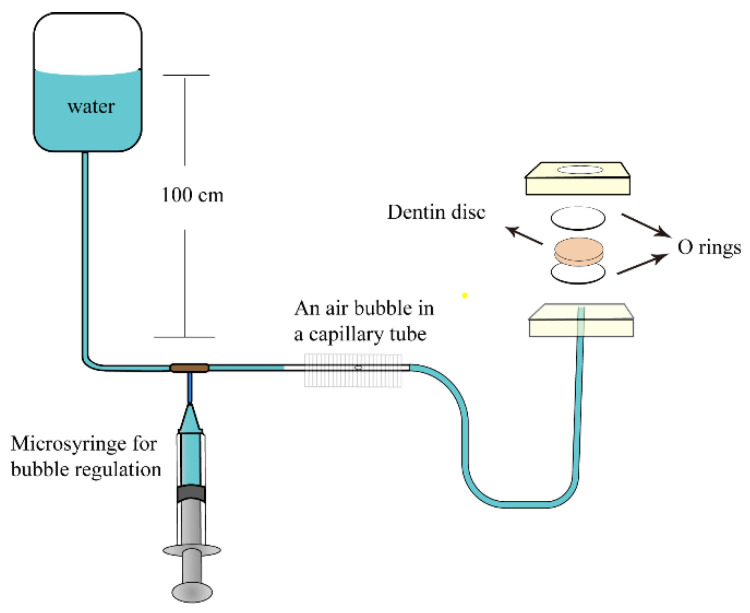
Schematic diagram of the dentin permeability.

The device for measuring dentin permeability was prepared according to the matrix described by Outhwaite and Pashley (Figure 3). The dentin specimen was placed in a split chamber with a pair of rubber “O” rings with an inner diameter of 6 mm and the effective transmission area was 0.2826 cm^2^. A container with deionized water provides 100 cm of H_2_O pressure for the dentin discs from the pulpal side to the occlusal side. The permeability was calculated using the following formulation: Lp = Jv/(A × t × P), where the Lp was the permeability of the dentin specimen (μL∙min^−1^ cm^−2^ cmH_2_O^−1^), Jv was the volume of deionized water passing through the dentin disc during the observation time (μL), A was the practical measurement area of the dentin specimen (cm^2^), t was the observation time (min), and P was the water pressure applied to the dentin specimen (cmH_2_O).

The whole device was filled with deionized water when the dentin specimen was installed. Then, a small bubble was injected through a microsyringe. After the bubbles remained stable for 3 min, the distance and time of movement were recorded. Before each test, the device seal was checked with a glass disc of the same size as the dentin discs to ensure that the device was completely sealed. Each dentin disc was measured thrice and the average value was obtained (n = 5). After acid etching, the dentin permeability was considered 100% and the relative dentin permeability was calculated for each group.

#### 2.8.4. FESEM

The dentin discs were fixed in 2.5% glutaraldehyde solution for 12 h at 4 °C and dehydrated in ascending ethanol concentrations. They were air-dried for 24 h at room temperature before sputter-coating with gold and observed under FESEM (Carl Zeiss, Jena, Germany) at 10 kV.

### 2.9. Statistical Analysis

All data were analyzed using SPSS 19.0 software (SPSS, Chicago, IL, USA). The normal distribution of all data was tested using the Kolmogorov–Smirnov test. The means and standard deviations for each group were calculated. One-way ANOVA was applied to statistically analyze the DC data. Two-way ANOVA was used to analyze the results of FWMH, Vickers microhardness and dentin permeability. Multiple comparison analysis was performed using the Tukey test. Statistical significance was set at 5% (*p* = 0.05).

## 3. Results

### 3.1. Structural Characterization of the Light-Curable Hydrophilic PU

**Figure 4:**
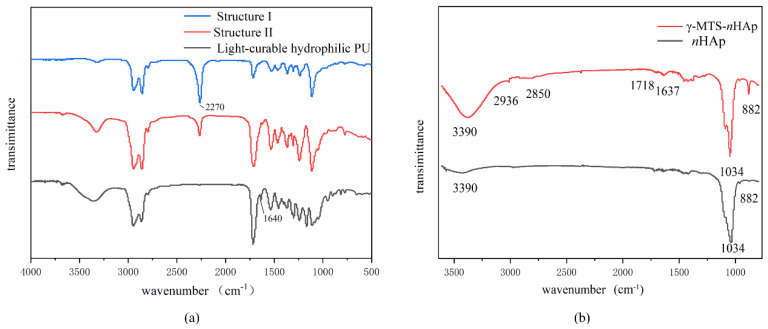
(**a**) The FTIR spectra of light−curable hydrophilic PU. (**b**) FTIR spectra of *n*HAp and *γ*−MTS−*n*HAp.

The FTIR spectra of light-curable hydrophilic PU were displayed in Figure 4a. In the first phase, the -OH absorption band disappeared at 3400 cm^−1^ and the stretching vibration of -NCO was observed at 2270 cm^−1^. Next, the signal intensity of -NCO (2270 cm^−1^) decreased significantly due to DMPA. After adding HEMA, the C=C stretching vibration appeared (1640 cm^−1^), whereas the -NCO absorption peak disappeared that was attributed to further polymerization.

### 3.2. Structural Characterization of γ-MTS-nHAp

Figure 4b illustrated the FTIR spectra of *n*HAP and *γ*-MTS-*n*HAp. The former exhibited the O–H bending vibration at 3390 cm^−1^ and phosphate bands of HAp at 1034 cm^−1^ and 964 cm^−1^. In addition to the above characteristic peaks, *γ*-MTS-*n*HAp showed a C=O stretching vibration (1718 cm^−1^) and bending vibrations of –CH_3_ (2936 cm^−1^), –CH_2_ (2850 cm^−1^) and C=C (1637 cm^−1^), which were characteristic peaks for *γ*-MTS.

### 3.3. DC

**Figure 5:**
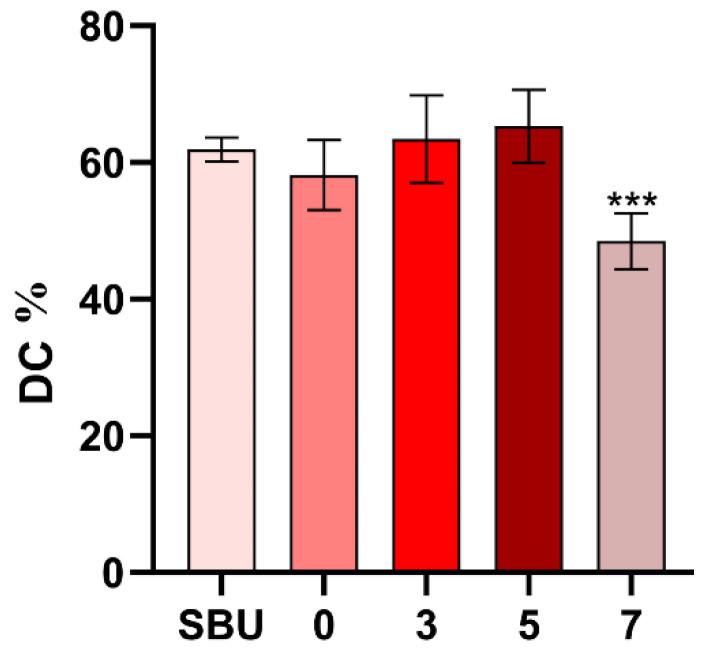
The degree of conversion of DDDEEKC-PU. *** *p* < 0.01.

The outcomes of DC were presented in Figure 5. DDDEEKC did not jeopardize the polymerization of the primers except the 7-PU group.

### 3.4. Vickers Microhardness

Table 1 illustrated the results of microhardness testing. Two-way ANOVA showed that mineralization time (*p* < 0.05) and DDDEEKC content (*p* < 0.05) significantly affected the dentin surface microhardness and interaction occurred between them (*p* < 0.05). All groups exhibited a significant increase after 28 days. The 0-PU group had the lowest value after 7 days, whereas the 5-PU (86.8 ± 2.18 MPa) and 7-PU (85.40 ± 3.61 MPa) groups had the highest value after 28 days.

### 3.5. XRD Findings

**Figure 6:**
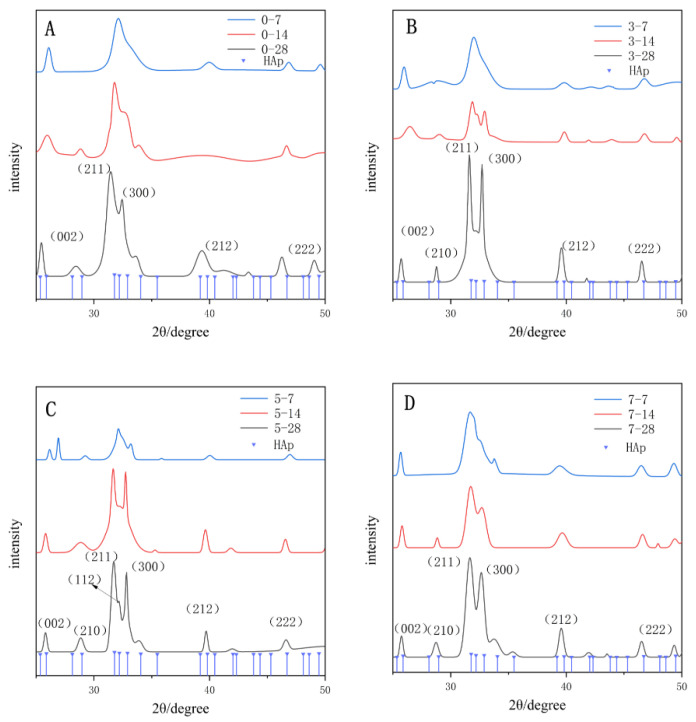
XRD results for the dentin discs mineralization of groups (**A**) 0-PU, (**B**) 3-PU, (**C**) 5-PU, (**D**) 7-PU for 7, 14 and 28 days.

Figure 6 showed the XRD characterization results after 7, 14 and 28 days for each group. After 7 days, a low single peak (002) was visible at 25.8° in all groups. The 5-PU and 7-PU groups had inconspicuous peaks at 32.8° (300), but no other derived peaks appeared.

After 14 days, characteristic peaks of HAp were observed in all groups at 25.8° (002), 31.8° (211), 32.8° (300), 39.2° (212) and 46.7° (222), corresponding to the standard PDF card (#09-0432). These results indicated the formation of HAp crystals. A small shoulder peak at approximately 32.2° (112) was even observed in the 5-PU group after 28 days. The 5-PU group also had the smallest FWMH (Table 2), confirming that the HAp had perfect crystallization.

### 3.6. Dentin Permeability

The dentin permeability result was presented as a percentage of the maximum permeability, assuming that the acid etching (0.119 ± 0.0180 μL∙min^−1^∙cm^−2^∙cmH_2_O^−1^) was equal to 100%. Table 3 showed the dentin permeability of each group. Even in the control group (0-PU), the permeability decreased to 20.81 ± 3.83% after 7 days due to resin tag blockage. Two-way ANOVA showed that mineralization time (*p* < 0.05) and DDDEEKC content (*p* < 0.05) affected the results and interactions between the two study factors were observed (*p* < 0.05). Dentin permeability decreased with increasing mineralization time in all groups. This result corresponded with the FESEM results. After 14 days, the permeability of the 7-PU group considerably reduced to 10.04 ± 1.02%, whereas the permeability of the 3-PU and 5-PU groups similarly decreased (*p* > 0.05). After 28 days, significant differences were noted among all groups (*p* < 0.05).

### 3.7. FESEM

**Figure 7:**
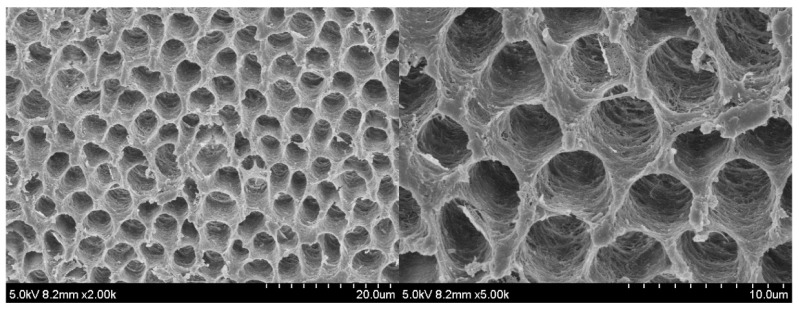
Scanning electron microscopy (SEM) images showing the dentin surface after acid etching.

As shown in Figure 7, dentin tubules were completely opened, and the collagen fibers were fully exposed after 30 s of acid etching. These features mimic the conditions of the dentin surface in clinical practice.

**Figure 8:**
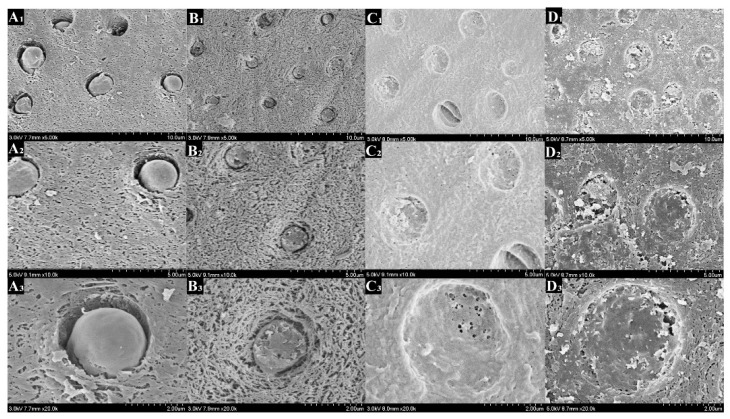
Scanning electron microscopy (SEM) images showing the DDDEEKC-PU treated dentin surfaces after 14 days of mineralization. The resin tags of the 0-PU(**A1**–**A3**) group had smooth surfaces that had gaps with the lumen of the dentin tubule. The 3-PU (**B1**–**B3**) group resin tags had granular and flaky mineralized deposits covering their surface with reduced gaps. The 5-PU (**C1**–**C3**) and 7-PU (**D1**–**D3**) groups had deposits that completely occluded the dentin tubules. The gaps were filled with deposits.

**Figure 9:**
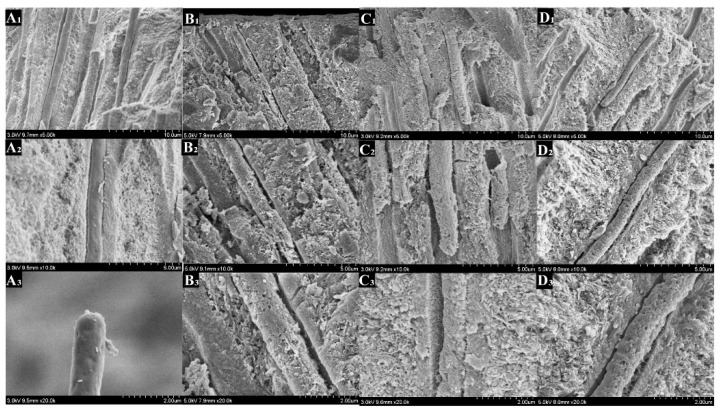
Scanning electron microscopy (SEM) images of dentin tubules from the cross view showing the DDDEEKC-PU treated dentin surfaces after 14 days of mineralization. The resin tags of the 0-PU(**A1**–**A3**) group had smooth surfaces and gaps visible with the lumen of the dentin tubules. The 3-PU group (**B1**–**B3**), 5-PU (**C1**–**C3**) and 7-PU (**D1**–**D3**) groups had needle-like, granular and spherical deposits on the surface of the resin tags.

**Figure 10:**
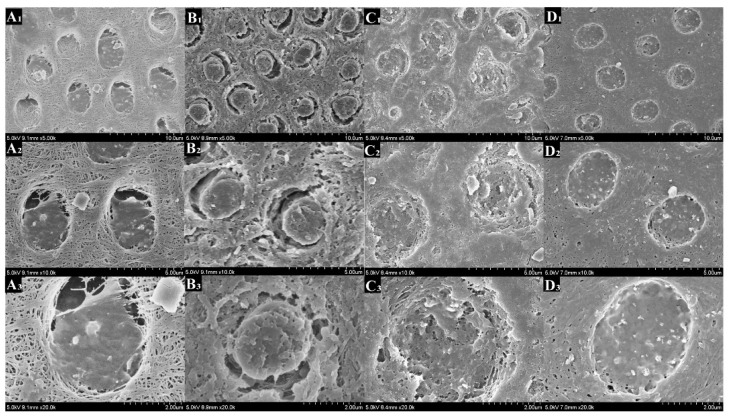
Scanning electron microscopy (SEM) images showing the DDDEEKC-PU treated dentin surfaces after 28 days of mineralization. The surfaces of the resin tags in the 0-PU(**A1**–**A3**) group did not change significantly compared with that in the 14th day (Figure 3). The 3-PU (**B1**–**B3**), 5-PU (**C1**–**C3**) and 7-PU (**D1**–**D3**) groups had many sheet-like deposits and fused in layers. All the gaps were filled.

**Figure 11:**
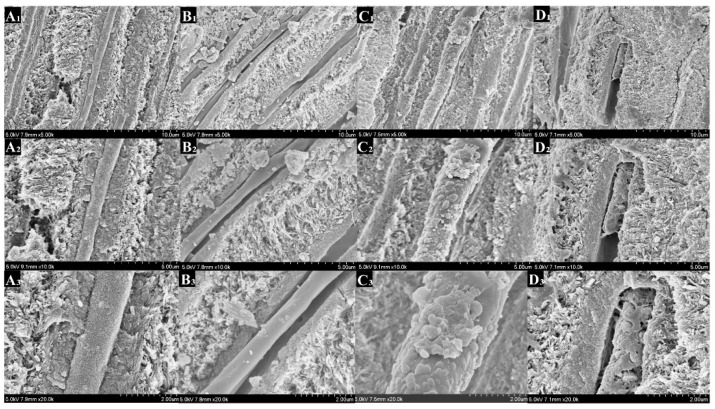
Scanning electron microscopy (SEM) images of dentin tubules from the cross view showing the DDDEEKC-PU treated dentin surfaces after 28 days of mineralization. Only scattered granular deposits appeared on the resin tags of the 0-PU(**A1**–**A3**) and 3-PU (**B1**–**B3**) groups. The 5-PU (**C1**–**C3**) group had many spherical and particle -like new mineral deposits on the surface. The deposits were dense and fused and the surface of the resin tags showed a ‘corn cob-like’ morphology. Although 7-PU (**D1**–**D3**) group had similar tags, resin hydrolysis appeared at most positions.

Figure 8, Figure 9, Figure 10 and Figure 11 showed the morphology of each group after mineralization. After 14 days, the resin tags of the 0-PU group filled dentin tubules with a smooth surface that had gaps with the lumen. In the 3-PU group, the granular mineralized deposits covered the resin tag surface, narrowing the gaps. The 5-PU group was similar to the 7-PU group that dentin tubules were filled with resin tags containing HAp deposits on the surface. After 28 days, all experimental groups, especially the 5-PU and 7-PU groups, had many sheet-like deposits that were fused in layers, whereas the 0-PU group did not change significantly.

On the cross section, the morphology resin tags of the 0-PU group were smooth and flat. The 3-PU and the 5-PU groups had needle-like and granular deposits. After 28 days, only scattered granular deposits appeared in individual sites of the 0-PU and 3-PU groups. The 5-PU group had many spherical and particle-like nascent mineral deposits on the surface that increased in size. The deposits were dense and fused; thus, the surface of the resin tags showed a corn cob-like morphology. Although the 7-PU group also exhibited similar morphology, resin hydrolysis occurred at most positions that disintegrated the resin tags.

## 4. Discussion

This study aimed to evaluate the effectiveness of dentin tubule filling and dentin mineralization among biomimetic mineralized hydrophilic polyurethane dentin primers. The results confirmed that dentin tubules are tightly filled with the novel dentin primer. Thus, both hypotheses were rejected.

The exposure of dentin tubules is one of the main causes of restoration bond failure and dentin hypersensitivity. According to Brännström and Åström’s hydrodynamic theory, blocking intra-dentinal tubular fluid movement via dentin tubule filling may reduce dentin hypersensitivity [19]. Several approaches have been used to occlude tubules, such as desensitizing toothpaste, laser re-solidification and remineralization [20]. Conventional desensitization agents are easily washed off by saliva in the variable oral environment if these agents cannot bind to the dentin surface stably. Moreover, it takes too long to close the dentin tubules and relief hypersensitivity symptoms by mineralization alone. At the same time, although polyanionic macromolecules can be directly used as primers to induce the remineralization of the hybrid layer, the retained polyanionic macromolecules are limited and the operation is complicated. In this study, the light-curable monomer PU formed a stable micromechanical locking junction with dentin to seal tubules instantaneously and the peptide DDDEEKC induced biomineralization to enhance the sealing effect. At present, few studies have focused on the double mechanism that associates the immediate sealing properties of primers with the subsequent induction of the peptide’s mineralization properties to fill dentin tubules and optimize the bonding surface.

Biomineralization is the formation of organic–inorganic complexes with specific orientations, hierarchical structures and special functions regulated by the organic matrix and biomolecules. Scholars have proposed classical nucleation theory and non-classical crystallization theory to explain the process. Classical crystallization theory proposes that biomineralization starts from atoms, ions and molecules, which can assemble gradually into clusters through the up-bottom approach. Clusters exist in a dynamic equilibrium of dissolution and growth until the crystal nucleus forms when their size achieves the critical threshold. Subsequently, the crystal nucleus grows further by attracting Ca^2+^ and PO_4_^3−^ forming hydroxyapatite crystals eventually. Crystal nucleation, which is completely dependent on free ions in solution, is referred to as homogeneous nucleation and the nucleation catalyzed by the residual amorphous phase is known as heterogeneous nucleation [21,22]. As detection techniques advances, there were more and more evidence that the crystalline formation does not necessarily follow the classical crystallization. Researchers subsequently proposed non-classical crystallization theory, which suggests that biomineralization is based on precursors, such as amorphous calcium phosphate and pre-nucleated clusters. These precursors aggregate into nanoparticles that penetrate collagen fibrils and form the metastable crystalline phase. Eventually, the metastable crystalline phase is transformed into hydroxyapatite crystals, completing the bottom-up mineralization [23]. However, natural NCPs cannot be extracted and purified easily in vitro, restricting their practical applications. Biomimetic peptides containing functional amino acid residues can partially mimic the functions of natural NCPs in promoting hydroxyapatite formation.

In this experiment, the peptide DDDEEKC was used as an NCP analog that was structurally and functionally similar to the native peptide segment SN15. This inexpensive peptide of low-molecular-weight has a strong affinity for HAp and can induce mineralization with no additional immunogenicity [24]. In the oral field, the amyloid protein-DDDEEK-coated Ti6Al4V screws showed better osteogenesis and osseointegration than HAp-sprayed screws [25]. The bioinspired DDDEEKC-decorated tannic acid can induce enamel remineralization and resist washing by acid and deionized water [26]. DDDEEKC-oligomeric procyanidins were applied as an in situ repair and bacteriostatic material on demineralized human enamel [27].

To confirm the stable prominence of DDDEEKC-PU, this work assessed dentin permeability using a hydrodynamic device that simulates pulp chamber pressure. According to Poiseuille’s law, dentin tubule diameter affects tubule fluid flow under constant pulpal internal pressure. Therefore, the dentin tubule diameter can be quantified by measuring the velocity of the dentin tubule fluid. The results of dentin permeability, together with FESEM results, can verify the filling of dentin tubules [28].

During the experiment, the bubble remained stationary for several periods rather than moving at a constant speed. This finding is consistent with the experimental phenomenon observed by Hiller et al. that short periods of measurement or discontinuous measurements are unable to judge dentin permeability accurately [29]. Therefore, the distance of bubble movement was measured for 5–10 min after 3 min of bubble stabilization as the evaluation criterion. Even in the control group (0-PU), although no HAp was observed by FESEM or XRD, the permeability decreased to 20.81 ± 3.83% after 7 days due to resin tag blockage. The permeability decreased in all groups as the mineralization time increased and the 5-PU and 7-PU groups had the lowest permeability at the same mineralization time. This result was consistent with the FESEM results.

However, one difference compared with the FESEM results was that the permeability of the 5-PU and 7-PU groups was similar, although the 7-PU group had resin hydrolysis. The permeability of the 7-PU group did not increase and even tended to decrease further due to incomplete hydrolysis existing only at the end of the resin tags close to the pulp chamber. Furthermore, a large amount of DDDEEKC promoted deposition that maintained the 7-PU group at a lower permeability.

DDDEEKC-PU also improve dentin bonding. Both light-curable hydrophilic PU and AA_60%_ contained hydrophilic -COOH or -COOK. AA_60%_ was polymerized into PU system to introduced more -COOH and -COOK. Here, -COOH and -COOK form hydrogen bonds with water molecules to restrict their diffusion, endowing the primer with excellent water absorption ability to block the water under the bonding layer and reduce the influence of water on the bonding layer while sealing dentin tubules.

**Figure 12:**
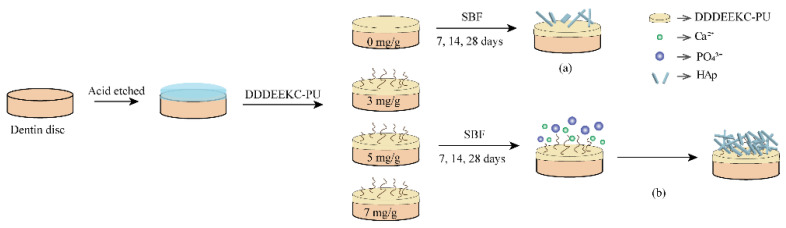
Schemas of remineralization process. (**a**) Hydroxyapatite formed in 0−PU group and (**b**) in 3−, 5−, 7−PU group. 0-PU develops nascent HAp through heterogeneous nucleation. Moreover, *γ*−MTS−*n*HAp also can absorb Ca^2+^ and PO_4_^3−^. In addition, in the experimental groups, the peptide DDDEEKC in the primer acted as NCP analog to induce mineralization.

In addition, during dentin bonding, both etch-and-rinse and self-etching adhesive systems dissolve HAp through acid etching to form a microporous structure allowing the adhesive resin to penetrate and create micromechanical interlocking. However, it also leads to the loss of minerals, decreasing mechanical properties and reducing the surface hardness at the bonding interface, especially in the etch-and-rinse system [30]. Biomimetic mineralization re-establishes the mineral composition of the adhesive interface by inducing the aggregation of Ca^2+^ and PO_4_^3−^, leading to HAp deposits within the hybrid layer. The HAp strengthens the micromechanical interlocking and restores the mineral content of the hybrid layer. In this study, the Vickers hardness of the dentin surface increased in all groups after 14 days, even in the control group. It was hypothesized that a few nuclei remained in the dentin after acid etching, which could form nascent HAp through heterogeneous nucleation in a remineralization environment. Moreover, *γ*-MTS-*n*HAP and AA_60%_ can absorb Ca^2+^ and PO_4_^3−^, contributing to the recovery of the surface hardness (Figure 12). However, no significant difference was noted between the 3-PU and the control groups after 14 days due to the low DDDEEKC content. The 5-PU and 7-PU groups had similar results after 14 and 28 days, indicating that the effect of DDDEEKC on hardness no longer appeared as the content reached 5 mg/g. The XRD results showed that the FWMH decreased and the diffraction intensity increased with increasing mineralization time and DDDEEKC content in all groups. The 5-PU group showed higher crystallinity and a more uniform HAp size after 28 days.

After 28 days, the 5-PU group had many spherical and particle-like nascent mineral deposits on its surface that increased in size. The deposits were dense and fused so that the surface of the resin tags showed a corn cob-like morphology. The nascent HAp on the resin tags optimizes their strength. Furthermore, it also improves the sealing ability of resin tags, reducing the influence of dentin tubule fluid on the bonding interface. Although the 7-PU group exhibited a similar morphology, resin hydrolysis occurred at most positions. Resin tags disintegrated into a porous structure. This finding was related to the low DC of the 7-PU group. Incomplete polymerization of the primer and significant hydrolysis of the resin tags resulted in morphological changes.

In the dentin adhesive system, the unpolymerized resin monomer becomes the vulnerable component of the hybrid layer. With external stress, saliva washing and oral temperature changes, the hybrid layer forms a permeable structure due to the dissolution of the monomer that increases water absorption and accelerates dentin collagen degradation [31]. In addition, the unpolymerized resin monomer is likely to induce streptococcal colonization, accelerate bacterial biofilm formation and increase the risk of secondary caries [32]. In this experiment, although HEMA and TEGDMA were added to dilute the PU substrate, the primer was still sticky. This property hindered free radical movement considerably in the free radical polymerization process. Therefore, DDDEEKC content should minimize the effect on DC. The PU matrix should form a tighter polymerization network and maintain the mechanical strength of the bonding interface.

In summary, the novel primer can seal the dentin tubules and induce the formation of hydroxyapatite on its surface. This property promotes mineralization and strengthens the hybrid layer. However, in future studies, improving dentin bonding durability by incorporating novel dentin primer with polyurethane adhesives needs further evaluation.

## 5. Conclusions

The light-curable hydrophilic PU was successfully synthesized and the optimal concentration of the peptide DDDEEKC was determined for the biomimetic mineralized hydrophilic polyurethane dentin primer, which can effectively fill dentin tubules through immediate sealing and subsequent mineralization. The biomimetic mineralized hydrophilic polyurethane dentin primer containing DDDEEKC offers great potential for treatment in dentin hypersensitivity and dental adhesive systems.

## Figures and Tables

**Table 1:** Vickers microhardness of the dentin surface after mineralization.

	0-PU	3-PU	5-PU	7-PU
7 days	58.32 ± 4.01 ^A,a^	60.92 ± 3.61 ^A,a^	64.97 ± 2.20 ^A,b^	70.11 ± 2.59 ^A,c^
14 days	61.45 ± 2.51 ^A,a^	65.13 ± 3.89 ^B,a^	70.23 ± 5.87 ^B,b^	72.90 ± 6.57 ^A,b^
28 days	70.60 ± 1.36 ^B,a^	80.28 ± 3.48 ^C,b^	86.8 ± 2.18 ^C,c^	85.40 ± 3.61 ^B,c^

Different lowercase letters in the same line indicate significant differences among groups (*p* < 0.05). Different uppercase letters in the same column indicate significant differences among groups (*p* < 0.05).

**Table 2:** The FWMH of the dentin surface after mineralization.

	0-PU	3-PU	5-PU	7-PU
7 days	0.78 ± 0.08 ^a,A^	1.20 ± 0.26 ^a,A^	0.86 ± 0.15 ^a,A^	0.76 ± 0.09 ^a,A^
14 days	1.11 ± 0.23 ^a,A^	0.77 ± 0.06 ^a,B^	0.74 ± 0.10 ^a,A^	0.81 ± 0.03 ^a,A^
28 days	1.09 ± 0.53 ^a,A^	0.46 ± 0.26 ^bc,B^	0.31 ± 0.12 ^b,B^	0.76 ± 0.03 ^ac,A^

Different lowercase letters in the same line indicate significant differences among groups (*p* < 0.05). Different uppercase letters in the same column indicate significant differences among groups (*p* < 0.05).

**Table 3:** The dentin permeability of the dentin discs after mineralization.

	0-PU	3-PU	5-PU	7-PU
7 days	20.81 ± 3.83 ^A,a^	17.35 ± 3.84 ^A,b^	13.21 ± 1.78 ^A,c^	13.62 ± 3.88 ^A,c^
14 days	17.22 ± 3.22 ^B,a^	12.56 ± 0.91 ^B,b^	13.33 ± 1.25 ^A,b^	10.04 ± 1.02 ^B,c^
28 days	18.68 ± 2.93 ^B,a^	14.2 ± 1.32 ^B,b^	11.07 ± 2.48 ^B,c^	7.58 ± 1.39 ^C,d^

Different lowercase letters in the same line indicate significant differences among groups (*p* < 0.05). Different uppercase letters in the same column indicate significant differences among groups (*p* < 0.05).

## Data Availability

The data will be shared on reasonable request to the corresponding author.
